# Treatment of Peripheral Nerves With Pulsed Radiofrequency: A Retrospective Analysis

**DOI:** 10.7759/cureus.79879

**Published:** 2025-03-01

**Authors:** Angela Haehnsen, Eckhard Mauermann, Konstantinos Kalimeris

**Affiliations:** 1 Anesthesiology, Triemli Municipal Hospital, Zurich, CHE

**Keywords:** interventional pain management, nerve blocks, neuromodulation, neuropathic pain, painful neuropathy, peripheral nerves, pulsed radiofrequency, refractory pain

## Abstract

Background: Pulsed radiofrequency (PRF) has evolved as a promising neuromodulative technique in chronic pain. Although it was initially used to treat spinal ganglia, it seems that it might also positively affect peripheral nerves. However, clinical evidence of its effect on peripheral nerves remains scarce.

Methods: In this retrospective study, we included patients with therapy-refractory chronic pain who received PRF treatment of peripheral nerves during an 11-month period in the pain center of a tertiary hospital in Zurich, Switzerland. A total of 17 treatments were analyzed. Pain scores, Global Impression of Change (GIC) scores, reduction of medication, and time to next infiltration up to three months after PRF were documented. Nine different peripheral nerves were treated.

Results: Pain scores were statistically lower at two weeks, one month, and three months after therapy. Overall, eight (47%) and five (29%) patients reported at least a 50% decrease in pain at one and three months, respectively. Of all patients, 12 (71%) reported an improvement in GIC at one and three months, while 10 (67%) could reduce or cease pain medications. PRF resulted in 14 (82%) patients not requiring a new infiltration for at least three months. However, in the presence of a psychiatric diagnosis, results were poorer.

Conclusions: Using PRF on peripheral nerves provided promising clinical results in terms of pain and impact of change in therapy-refractory cases. PRF might be a useful tool in pain medicine when the effects of nerve blocks are not sustainable. Further research is warranted.

## Introduction

Chronic pain can often be caused by peripheral neuropathies. Trauma and operation are among the causes, while refractory cases present dramatically poorer health and disability [1]. Management of this type of chronic pain can be problematic. Targeted diagnostic blocks, especially with the use of ultrasound, can aid in identifying the target nerve site to proceed to therapeutic blocks [2]. However, therapeutic infiltrations often show only a short-acting effect and must be repeated often during a long period of time, even when anti-neuropathic medications are concomitantly used [3]. This is obviously far from an ideal therapeutic plan, creating a pragmatic necessity for more sustainable therapies.

Pulsed radiofrequency (PRF) is an interventional pain therapy that was initially launched as a neuromodulative technique in the dorsal root and trigeminus ganglia [4,5]. Its mechanism of action is not fully understood; however, a number of functional changes have been experimentally shown, not only on the peripheral site but also on the corresponding dorsal root ganglion and spinal segment, resulting in improved pain and reversal in some instances of neuronal injury [6,7]. Because PRF is essentially the application of an electromagnetic field on the nerves at no more than 42°C, it is considered a nondestructive technique that would be more attractive than cryotherapy or thermoablation for use on peripheral nerves [8]. However, evidence on the efficacy and duration of effect remains scarce; it remains largely unknown if PRF yields meaningful results in pain therapy and in which nerves it could be applied. In this retrospective analysis, we investigate the effect of PRF on peripheral nerves in patients with therapy-refractory pain syndromes necessitating repetitive therapeutic infiltrations.

## Materials and methods

Study design and patients

This is a descriptive retrospective study for the use of PRF in chronic peripheral nerve pain during a period of 11 months. Ethical approval was granted for this study by the appropriate institutional review board (EK Zuerich; Approval Number: 2024-00755; dated: 06.08.2024). We included adult patients (>18 years) who received PRF therapy for chronic pain on peripheral nerves between April 2023 and February 2024 at the pain center of a tertiary public hospital (Zurich City Hospital, Zurich, Switzerland). Only patients who had signed a general consent form for research were included; those without such a form or not wishing to be involved in research analyses were excluded. Chronic pain was defined as moderate to severe pain with a duration of three months or more. We excluded patients receiving PRF on medial branches (considered as spinal intervention) and in cases in which legal processes for invalidity were active. The existence of psychiatric disease in the medical record (depression, personality disorder, etc.) was not an exclusion criterion but was noted for subgroup analyses. Patients with psychiatric disorders received psychiatric or psychological treatment/support. Following the biopsychosocial model, psychological support was recommended to all patients, in whom an important psychological burden was recognized during initial assessment, as part of our clinical routine [9].

Pulsed radiofrequency

Patients were instructed not to change their usual (pain) medications before the PRF procedure but were free to do so afterwards according to their pain level. No sedation was used. PRF was performed under ultrasound guidance (Sonosite PX Stand, Fujifilm Sonosite Inc., Bothell, WA, USA) using a special radiofrequency needle (22/20G, length 50 mm or 100 mm, with active tip of 5, 7, or 10 mm, according to the size of the nerve) and a corresponding thermocouple electrode 50 mm or 100 mm (both from Inomed Medizintechnik GmbH, Emmendingen, Germany). The RF generator used was LG2 Lesion Generator (Inomed Medizintechnik GmbH, Emmendingen, Germany). Monopolar PRF was used. A neutral plate electrode was placed on the skin of the patient in proximity to the nerve blocked. The blocks and the PRF were performed by two experienced physicians and tutors in ultrasound (AH and KK). The procedure involved a careful sonographic analysis of the anatomy and the planning of the needle trajectory to place the needle as perpendicularly as possible to the target nerve. After local anesthesia of the skin and tissues with procaine 1%, the PRF needle was brought - usually using the short-axis/in-plane approach - perpendicularly to the nerve. If required, hydrodissection was used to locate the tip of the needle. If deemed necessary, the absence of motor stimulation was confirmed at 2Hz up to 2.0V (for example, not for tibial nerve); otherwise, this step was omitted. Sensory stimulation was performed at 50Hz up to 1.0V, seeking an appropriate answer (pain/tingling/pressure/paresthesia) in the painful area. Therapy was then performed, consisting of two to three cycles of PRF of 60-70V at 42°C for 2.5 minutes in adjacent positions, with a pause of 30 seconds between the cycles [10-12]. Although we initially used temperature-adjusted therapy, we continued with voltage-adjusted PRF and higher voltage in most patients because 42°C was otherwise not promptly reached. To this time, PRF is not a standardized therapy in pain medicine, but rather used as ultima ratio in difficult cases. Therefore, it may be understandable that variable protocols are used in clinical practice and also in research protocols. For example, in a comprehensive review of the use of PRF on occipital nerve, it is shown that variable protocols are used [11]. We decided to use two to three cycles, depending on the anatomy of the nerve on ultrasound, whether the nerve was reached well by the needle tip, and how close to the skin the nerve was. There is also currently no consensus on the optimal voltage, this is why we began therapy with 60V. In some cases, however, therapy did not reach 42°C, which signifies a successful treatment, and we had to increase the voltage to 70V, which is justified by the more superficial location of some small nerves. In the cases where the temperature was not reached from the beginning, we chose three cycles instead of two. Other researchers published a range of voltage rather than a single value [12]. During therapy, the correct position of the needle tip was repeatedly confirmed by ultrasound to decrease the risk of needle dislocation. Afterwards, 2-5 ml of ropivacaine 0.3-0.5% (ROPIVACAIN SINTETICA) with usually a small amount of steroid (dexamethasone; MEPHAMESON®) or, more rarely, triamcinolone (KENACORT®-A) was given through the cannula (not in subcutaneous nerves). The cannula was flushed with normal saline and then retrieved.

Follow-ups and endpoints

Patients were contacted, as usual in our clinical practice, after two weeks, and at one and three months, to identify any possible adverse effects and to assess pain control. Patients were clearly instructed to contact the pain center sooner if pain increased and to ask for a new infiltration if they felt that one was needed. A questionnaire was filled out at three months, asking for the result on pain intensity (numerical rating scale (NRS), 0- 10), the patient global impression of change scale (GIC, 1-7; 1 = very much better, 2 = much better, 3 = little better, 4 = no change, 5 = little worse, 6 = much worse, 7 = very much worse), and if pain medications were reduced or ceased after the therapy. We also documented the time required until a new infiltration of the involved nerve was performed.

Statistical analysis

Categorical data are presented as absolute numbers (percentage), and continuous data are presented as median (range) for descriptive variables. Potential differences in NRS and GIC to baseline were examined by paired Mann-Whitney U-test, respectively, with values presented as median (Q1, Q3). As this is an explorative study, no p-value corrections were made for multiple testing, and p < 0.05 was considered to be statistically significant. No sample size estimation was performed in this retrospective study as no information regarding the expected effect size is known.

## Results

A total of 17 PRF interventions (14 patients) were included. The median age was 57 years (range: 23-84), and 11 (79%) were female. Patients were on pain medications (e.g., paracetamol, metamizole, opiates, non-steroidal anti-inflammatory drugs, antiepileptics, or antidepressants) without sufficient pain control or had stopped them because of lack of effect or adverse effects. Pain diagnosis, the nerve treated, the type of pain, the presence of psychiatric diagnosis, and pain scores after PRF are shown for each patient in Table 1. Nine different peripheral nerves were treated, two patients were treated twice with PRF (in a more than three-month interval) on the same nerve, and one patient had separate interventions on two different nerves. Five patients had a psychiatric diagnosis in their medical history.

**Table 1:** Nerves treated, variables of the treatments, and pain scores. ILH-ILN: iliohypogastric and ilioinguinal nerves; LFCN: lateral femoral cutaneous nerve; NRS: numerical rating scale; PD: psychiatric diagnosis (Y: yes; N: no); PHN: postherpetic neuralgia.

Patient No.	Nerve	Diagnosis	Pain phenotype	PD	NRS before	NRS 2 weeks	NRS 1 month	NRS 3 months
1	Genicular nerves	Knee osteoarthritis	Mixed	N	7	1	1	2
2	Saphenous nerve	Postoperative pain	Neuropathic	N	5	5	6	6
3	Sural nerve	Posttraumatic-postoperative pain	Mixed	N	7	6	2	6
4	Genicular nerves (2^nd^)	Knee osteoarthritis	Mixed	N	7	3	3	7
5	Sural nerve (2^nd^)	Posttraumatic-postoperative pain	Mixed	N	7	4	2	3
6	Intercostal nerve	PHN	Neuropathic	N	4	0	1	3
7	Medial plantar nerve	Posttraumatic-postoperative pain	Mixed	Y	6	6	4	4
8	ILH-ILN	Postoperative pain	Mixed	Y	6	2	1	1
9	LFCN	Meralgia paresthetica	Neuropathic	Y	4	1	3	4
10	Genitofemoral and LFCN	Neuropathic pain	Neuropathic	Y	7	1	7	3
11	Genitofemoral nerve	PHN	Neuropathic	N	6	3	3	4
12	LFCN	PHN	Neuropathic	N	7	5	6	6
13	LFCN	Postoperative pain	Neuropathic	Y	9	7	7	6
14	Sural nerve	Posttraumatic-postoperative pain	Mixed	Y	4	4	4	4
15	Genitofemoral nerve	Postoperative pain	Neuropathic	N	6	1	2	6
16	Major occipital nerve	Occipital neuralgia	Neuropathic	Y	7	6	7	7
17	Major occipital nerve	Postherpetic	Neuropathic	N	9	5	5	2

**Figure 1:**
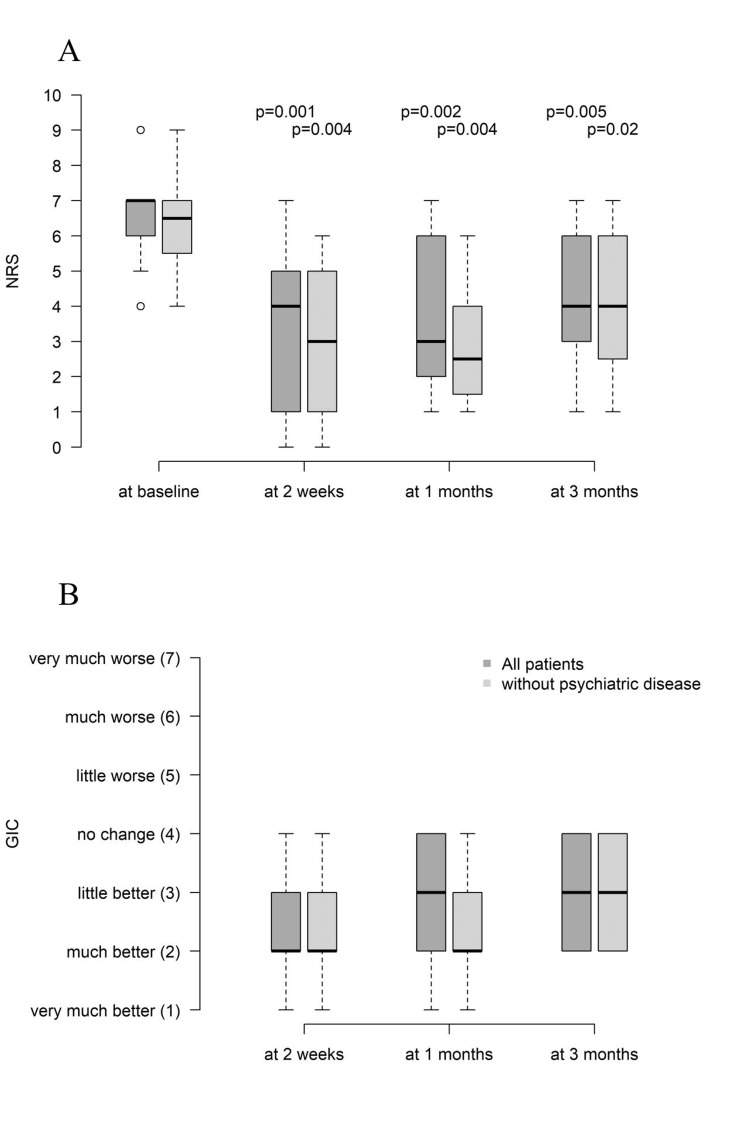
Pain and GIC scores. A: Numerical rating scale (NRS) scores over time after pulsed radiofrequency (PRF). B: Global Impression of Change (GIC) scores after PRF.

Figure 1 shows box plots for NRS pain and GIC for all patients (dark gray) as well as for those patients without documented psychiatric disease (light gray). Despite the small sample size, pain reduction in comparison to baseline was statistically significant at all time-points (n = 17; two weeks: 4 (Q1, Q3: 1, 5) vs. 7 (Q1, Q3: 6, 7); one month: 3 (Q1, Q3: 2, 6) vs. 7 (Q1, Q3: 6, 7); three months: 4 (Q1, Q3: 3, 6) vs. 7 (Q1, Q3: 6, 7)) and even nominally lower when excluding patients with documented psychiatric disease. It seems possible that patients with psychiatric disease are less likely to benefit from PRF (NRS baseline: median = 7.0 (Q1, Q3: 6.0 - 7.0); NRS at two weeks: median = 6.0 (Q1, Q3: 4.0 - 6.0); NRS at four weeks: median = 7.0 (Q1, Q3: 4.0 - 7.0); NRS at three months: median = 4.0 (Q1, Q3: 4.0 - 6.0)). Table 2 shows NRS pain and GIC scores as absolute values as well as the number and percentage of patients with a significant (≥50%) decrease in NRS, as well as the number of patients with GIC of 3 or less. In 10 (67%) out of the 15 cases in which pain medications were used prior to PRF, PRF resulted in reduction or cessation of the pain medication. Additionally, 14 (82%) patients did not require a therapeutic infiltration for at least three months.

**Table 2:** Pain and GIC scores. NRS: numerical rating scale; GIC: Global Impression of Change.

	Before	2 weeks	1 month	3 months
NRS	7 (IQR 6, 7)	4 (IQR 1, 5)	3 (IQR 2, 6)	4 (IQR 3, 6)
Number (%) of patients with ≥ 50% decrease in NRS	-	8 (47%)	8 (47%)	5 (29%)
GIC	-	2 (IQR 2, 3)	3 (IQR 2, 4)	3 (IQR 2, 4)
Number (%) of patients with GIC ≤ 3	-	14 (82%)	12 (71%)	12 (71%)

## Discussion

This study provides initial findings that using PRF on peripheral nerves could have a clinical meaning in chronic pain resulting from and/or transmitted by a peripheral nerve. We initiated the use of PRF in our center as an attempt to provide a better solution to patients with refractory pain despite repetitive infiltrations. As commonly encountered in chronic pain, the ratio of female patients was also higher among our patients [13]. Although evidence for the use of PRF on peripheral nerves is lacking at present, we decided to implement PRF as ultima ratio for these refractory cases [8]. Considered as such, PRF showed rather satisfactory results on pain, even three months after the intervention. Importantly, most patients reported a positive impression of change, and 82% of them did not require another therapeutic intervention for the next three months. Both findings could be interpreted as reaching a rather compensated pain state and satisfaction. We could also show that PRF resulted in reducing or stopping the pain medications, which might be interpreted as a less subjective measure of efficacy.

As a non-destructive, neuromodulative technique, PRF presents some important advantages; first, as it does not cause loss of function, it can be used on peripheral nerves innervating the skin without causing hypesthesia. Second, it can be applied on mixed nerves too, preserving motor function and enabling muscle activation and training. The above makes PRF more attractive for the treatment of peripheral nerves, compared to neurodestructive techniques, such as cryoablation. Cryoablation has shown promising initial results in terms of efficacy on peripheral nerves [14]; however, a major disadvantage of cryoablation is loss of function, which can be counterproductive in cases where exercise is important to improve functionality and prevent muscle loss. Hypesthesia is also an important drawback of applying extreme cold to peripheral nerves; PRF does not cause such issues.

Third, PRF seems to not cause neurinomas, which is a possible complication of thermoablative techniques [15]; indeed, none of our patients presented new neuropathic symptoms. Thermoablation seems to be effective in peripheral neuropathies; however, this therapy is accompanied by loss of function and skin hypesthesia and carries the risk of neurinoma formation, which can cause troublesome persistent pain [16]. Moreover, the application of 80°C near the skin can cause burns, which renders thermoablation a less attractive choice in superficial nerves.

Lastly, it can be applied at sites where a continuous block or a peripheral nerve stimulator would not be practical and without the need for a catheter [17,18]. It is nonetheless more time-consuming than a plain infiltration, requires expertise in the use of ultrasound, and has a higher cost. To what extent this is compensated by the decreased need for new interventions or the reduction of pain medications remains to be investigated.

Another finding of this study is the likely impact of psychiatric comorbidities on the response to treatment of chronic pain, which is, however, not striking but rather well documented in pain literature [19]. Although this study was not designed to address this issue specifically and the number of patients is limited, our clinical impression that these patients respond less well to pain therapies was confirmed.

We would additionally like to point out some considerations and technical notes. Up to this present, the technique of PRF on peripheral nerves is not standardized [20]. For instance, it is uncertain whether local anesthesia around the nerve before therapy affects the efficacy of the therapy or if saline should be used instead. In our experience, omitting local anesthesia near the nerve does not cause any significant discomfort; the patient feels solely the pulsation in the treated area. We believe that this might be important to consider in future studies. Second, despite the questionable role of steroids, regarding both its prophylactic use against neurinoma formation and its contribution to the therapeutic effect, we felt better to administer steroids in these refractory cases [21,22]. Third, we found that holding the needle stable and re-checking with ultrasound if the needle tip with the electrode still lies correctly against the nerve was necessary in superficial nerves (e.g., sural) due to the minor grasp of the needle in the tissues. Lastly, we initially noticed a delay to achieve 42°C when using the temperature-regulated PRF program, especially in superficial nerves or when more volume for hydrolocalization was used; this is why higher voltage with the voltage-regulated program (70V) was necessary in most cases. We feel that this might be an important contribution to the current discussion on optimal parameters for PRF [23].

Despite its novel approach and promising results, this retrospective study has certain limitations. As might be expected in a retrospective analysis of a novel application, not all methods were standardized; for example, the administration and dose of steroids, the cycles of PRF, and the voltage-regulated vs. temperature-regulated procedure. This variability in PRF protocols as well as drugs used might reflect the complexity of clinical reality; however, it limits the strength of the study. Additionally, the retrospective nature of the study, the small number of patients, the variable pain conditions, and the short follow-up weaken the impact of the study. These limitations could help optimize the design of future randomized clinical trials on PRF.

## Conclusions

We retrospectively analyzed the use of PRF on several peripheral nerves in chronic pain syndromes in a pragmatic clinical setting. We innovated using PRF on peripheral nerves because many patients in our pain center could not achieve satisfactory pain relief, despite the good response to repetitive blockades of peripheral nerves. Our findings suggest that PRF shows promising efficacy on peripheral nerves in the state of chronic pain, both in terms of pain and the impact of pain on life. However, because of the retrospective nature of the study, these results should be interpreted carefully. Future research should prospectively analyze the effect of PRF on peripheral nerves.
